# WT1: The Hinge Between Anemia Correction and Cancer Development in Chronic Kidney Disease

**DOI:** 10.3389/fcell.2022.876723

**Published:** 2022-04-06

**Authors:** Wen-Chin Lee, Chien-Hua Chiu, Tian-Huei Chu, Yu-Shu Chien

**Affiliations:** ^1^ Division of Nephrology, Department of Internal Medicine, Kaohsiung Chang Gung Memorial Hospital and Chang Gung University College of Medicine, Kaohsiung, Taiwan; ^2^ Medical Laboratory, Medical Education and Research Center, Kaohsiung Armed Forces General Hospital, Kaohsiung, Taiwan

**Keywords:** Wilms’ tumor 1, anemia, cancer, chronic kidney disease, hypoxia-inducible factor-prolyl hydroxylase inhibitor

## Abstract

Hypoxia-inducible factor-prolyl hydroxylase inhibitors (HIF-PHIs) emerge as promising agents to treat anemia in chronic kidney disease (CKD) but the major concern is their correlated risk of cancer development and progression. The Wilms’ tumor gene, *WT1*, is transcriptionally regulated by HIF and is known to play a crucial role in tumorigenesis and invasiveness of certain types of cancers. From the mechanism of action of HIF–PHIs, to cancer hypoxia and the biological significance of WT1, this review will discuss the link between HIF, WT1, anemia correction, and cancer. We aimed to reveal the research gaps and offer a focused strategy to monitor the development and progression of specific types of cancer when using HIF–PHIs to treat anemia in CKD patients. In addition, to facilitate the long-term use of HIF–PHIs in anemic CKD patients, we will discuss the strategy of WT1 inhibition to reduce the development and progression of cancer.

## Introduction

Anemia is a common complication of chronic kidney disease (CKD) (Stauffer and Fan, 2014; Sofue et al., 2020). It causes reduced quality of life and increased morbidity and mortality in CKD patients (Coresh et al., 2007; Cases et al., 2018; Lee et al., 2018). Anemia in CKD is caused by many factors, including inadequate production of erythropoietin, functional iron deficiency, chronic inflammation, metabolic acidosis, hyperparathyroidism, dietary deficiency of folic acid and vitamin B12, and the side effects of concurrent medications (Babitt and Lin, 2012; Gluba-Brzozka et al., 2020). In spite of its complex pathogenesis, erythropoiesis-stimulating agents (ESA) have improved the quality of life of patients, reduced anemia-associated cardiovascular morbidity and the requirement for blood transfusion (Stone et al., 1988; Drueke et al., 2006; Finkelstein et al., 2009; Lewis et al., 2011). Despite the clinical success of current injected ESA, several large studies have established that supraphysiologic dosing of ESA is associated with increased risk of cardiovascular events, vascular access thrombosis, and overall mortality (Szczech et al., 2008; Solomon et al., 2010).

Cardiovascular complications and safety concerns from current injected ESA have led to the development of alternative strategies for the treatment of renal anemia. One of the most promising approaches is the development of hypoxia-inducible factor-prolyl hydroxylase inhibitors (HIF–PHIs), which offer a more consistent physiological level of erythropoietin (EPO) to stimulate red blood cell production. Despite the promising data from clinical trials of HIF–PHIs on anemia correction in CKD patients, the increased HIF raises the concern of the cancer risks. It is known that hundreds of genes induced by hypoxia in an HIF-dependent manner encode proteins that play key roles in many aspects of cancer biology including proliferation, cell survival, epithelial-to-mesenchymal transition (EMT), angiogenesis, invasion and metastasis (Talks et al., 2000). Notably, a substantial proportion of these genes are regulated by the Wilms’ tumor gene (*WT1*) (Zhang et al., 2005; Wagner et al., 2008; Meyer et al., 2019; Le et al., 2020).Besides, *WT1* is critically regulated by HIF and plays a crucial role in tumorigenesis and metastasis. In this review, we will discuss the mechanisms of action, the outcome of the clinical trials, and the theoretical concerns regarding HIF-PHIs and malignancies. We will discuss HIF-PHIs and malignancies from the perspective of WT1.

## HIF-PHIs as a Promising Treatment for Anemia in CKD

### Mechanism of Action of HIF–PHIs

HIF, consisting of an oxygen-sensitive α-subunit and a constitutively expressed β-subunit, is a heterodimeric transcription factor responsible for activating the expression of EPO and genes involved in iron metabolism (Rankin et al., 2007; Kapitsinou et al., 2010; Kobayashi et al., 2016). HIF prolyl hydroxylase (HIF-PHD) enzymes affect the stability of the α subunit of HIF by promoting post-translational hydroxylation in an oxygen-dependent manner. HIF-PHIs temporarily inhibit PHD catalysis and contribute to a transient increase in HIF expression, regulating the function of many genes, including EPO, EPO receptor, proteins promoting iron absorption, iron transport, and heme synthesis (Bernhardt et al., 2010; Provenzano et al., 2016).

### Clinical Outcomes of HIF-PHIs in CKD

HIF-PHIs stimulate erythropoiesis in a dose-dependent manner and have consistently shown clinical efficacy in patients with anemia of non-dialysis-dependent and dialysis-dependent CKD in phase II and III studies. The HIF-PHI roxadustat, orally administered three times a week for 8 weeks, effectively corrected hemoglobin levels in a small double-blinded, placebo-controlled phase III study in China (Chen et al., 2019), and in a two-arm, randomized, open-label study in Japan (Akizawa et al., 2020). Preliminary results were comparable to those of darbepoetin alfa in a 52-weeks, randomized, open-label study in Japan (Akizawa et al., 2021). Besides, HIF-PHI administration in CKD patients was associated with an increase in total iron binding capacity in most phase II and III studies (Pergola et al., 2016; Akizawa et al., 2019). A comprehensive review on the clinical trial data of well-investigated HIF-PHIs has recently been published (Haase, 2021). There are four compounds being licensed for marketing in Asia. An investigation on their long-term safety, including the occurrence and progression of cancer in extended trials, and a post-marketing analysis are yet to be performed.

### Theoretical Concerns on HIF-PHIs and Malignancies

In addition to promoting erythropoiesis, the HIF pathway is essential for cellular survival under hypoxic conditions and regulates an array of biological processes, including cell growth and differentiation, angiogenesis, vascular tone, and metabolic processes (Semenza, 1999; Kewley et al., 2004; Wenger et al., 2005). The major concern is its effects on tumor growth and invasion as well as resistance to therapeutic agents. Activation of HIF-1α and HIF-2 has been shown to increase tumor survival in colorectal and breast cancers through different mechanisms (Kaidi et al., 2006; Choudhry et al., 2015). In addition, activation of HIF pathway has been reported to be associated with tumor aggressiveness, invasion, and metastasis through the c-Myc pathway in osteosarcomas (Yoo et al., 2011). It is also known to promote EMT in pancreatic cancer (Yang et al., 2016). In hematologic malignancies, overexpression of HIF-1α has been reported in acute myeloid leukemia (AML), acute lymphoblastic leukemia (ALL) and chronic myeloid leukemia (CML). HIF-2α overexpression has been demonstrated in both AML and ALL (Deeb et al., 2011; Frolova et al., 2012; Zhang et al., 2012; Forristal et al., 2015). Furthermore, roxadustat has been reported to increase the incidence of lung cancer in male mice and breast cancer in female mice compared with that in the control group (Beck et al., 2017). This evidence raises the theoretical concerns regarding HIF-PHIs and malignancies. Cancers currently known to be associated with the activated HIF pathway are summarized in Table 1.

**TABLE 1:** Cancers associated with the activated HIF pathway.

Cancers	Models	Species	References
Breast	Cell lines and patients	Human	Choudhry et al. (2015)
Colon	Cell lines	Human	Kaidi et al. (2006)
Lung	Animal	Mice	Beck et al. (2017)
Pancreas	Patients	Human	Yang et al. (2016)
Osteosarcoma	Cell lines	Human	Yoo et al. (2011)
Leukemia	Patients	Human	Deeb et al. (2011)

## The Link Between WT1 and Cancer

The *WT1* gene, located at chromosome 11p13 (Call et al., 1990), encodes for 10 exons and generates a 3 kb mRNA. There are two major alternative splicing events. These include splicing of exon 5 (17 amino acids), and of a stretch of nine nucleotides (three amino acids, lysine, threoine, and serine (KTS)) in the 3’ end of exon 9. Alternative splicing of these two sites results in four different protein isoforms designated A, B, C and D, representing the presence or absence of exon 5 and KTS insert, respectively. Under normal physiological conditions, the expression of KTS(+)/KTS(-) ratio is maintained at approximately 2:1 (Haber et al., 1991). The N-terminal domain of *WT1* is comprised of proline-glutamine-rich sequences and is critical for the transcriptional regulatory function of WT1. The C-terminal domain of *WT1* is composed of four zinc fingers, which allow binding to target DNA sequences but are also involved in RNA and protein interactions. Through the C-terminal half of the protein, *WT1* has been reported to be a potent transcriptional regulator targeting genes responsible for cellular growth and metabolism (Menke et al., 1998; Yang et al., 2007).

WT1 in concert with a variety of genes and proteins plays important roles in tumorigenesis and cancer metastasis. It is known to transcriptionally activate the proto-oncogene, c-Myc in human leukemic K562 cells and in several human breast cancer cell lines (Han et al., 2004). By protein-protein interactions, WT1 interacts with p53 and modulates their ability to transactivate their respective targets (Maheswaran et al., 1993). The clinical significance of the interaction between WT1 and p53 has been demonstrated in ovarian cancers (Carter et al., 2018). In addition, in solid tumors, WT1 activation has been shown in tumors originating from tissues that do not express WT1 in adults. The role of WT1 in controlling the balance between the mesenchymal and epithelial state of the cells might provide a critical link between WT1 and EMT, which is a key process for the metastasis of carcinomas. The two better studied major roles of WT1 in cancer development and metastasis are discussed below.

### *WT1* as an Oncogene

*WT1* was originally discovered as a tumor suppressor because of its loss-of-function mutations in a subset of pediatric renal neoplasms, known as nephroblastomas or Wilms’ tumors (Huff et al., 1991). It is well known that *WT1* is fundamental to mammalian organ development, including blood vessels, heart, spleen, liver and genitourinary system (Kreidberg et al., 1993; Herzer et al., 1999; Moore et al., 1999; Ijpenberg et al., 2007). On the other hand, although *WT1* behaves as a tumor suppressor gene in Wilms’ tumors, increasing data suggest a role for *WT1* as an oncogene in both leukemia and solid tumors (Osaka et al., 1997; Sera et al., 2008; Desmedt et al., 2009; Brett et al., 2013). These accumulating data has been summarized in comprehensive reviews (Yang et al., 2007; Huff, 2011; Chau and Hastie, 2012).

Compared with normal human tissues, *WT1* is expressed at a rather high level in various malignancies including ovarian (Hylander et al., 2006; Yamamoto et al., 2007; Andersson et al., 2014), breast (Loeb et al., 2001; McGregor et al., 2018), uterine (Coosemans et al., 2011; Guntupalli et al., 2013), lung (Oji et al., 2002; Hayashi et al., 2012), colon (Koesters et al., 2004; Bejrananda et al., 2010) cancers and malignant pleural mesothelioma (Cedres et al., 2014). In breast cancer, WT1 upregulates the expression of human epidermal growth factor receptor 2 (HER2), leading to estrogen-independent tumor growth and anti-estrogen resistance. Silencing of *WT1* inhibits the growth of MCF-7 cell line (Navakanit et al., 2007; Nasomyon et al., 2014). HER2 has been shown to upregulate WT1 expression through the AKT signaling pathway, promoting breast cancer cell proliferation and inhibiting cellular apoptosis (Tuna et al., 2005). In lung cancer, there is a positive feedback loop between WT1 and AKT-1. Cisplatin treatment downregulates the WT1 expression through the PI3K/AKT signaling pathway (Wang et al., 2013).

Although the molecular mechanisms that account for the increased expression of *WT1* in these cancers are not fully characterized, it has been reported that the proximal *WT1* promoter contains a hypoxia-responsive element (HRE), which is a binding site of HIF-1 (Wagner et al., 2003). Considering the relatively low oxygen tensions in rapidly growing tumors (Hockel and Vaupel, 2001), it would be reasonable to speculate that intratumoral hypoxia could lead to enhanced HIF expression, which transcriptionally activates *WT1* in these cancers. Supporting this perspective, the upregulation of both HIF1α and *WT1* has been reported in patients with myelodysplastic syndrome (MDS) or acute leukemia (Rosenfeld et al., 2003; Mpakou et al., 2021).

### WT1 Regulates the EMT

Despite mounting evidence demonstrating high levels of WT1 expression in leukemia and solid tumors as described above, the exact functional implications of increased WT1 expression in tumorigenesis are not fully understood. Nevertheless, WT1 has been shown to regulate cell proliferation, apoptosis, and blood vessels formation (Hartkamp and Roberts, 2008; Scholz et al., 2009), which all are well known biological processes leading to tumorigenesis when go awry. Furthermore, WT1 is known to control the cell transition between the mesenchymal and epithelial states by transcriptionally regulating major EMT mediators Snail (*Snai1*) and E-cadherin (*Cadh1*) during embryonic development (Martinez-Estrada et al., 2010). Uncontrolled EMT is a hallmark of various pathologies, including cancer, while disruption of mesenchymal-to-epithelial transition has been associated with a number of developmental abnormalities (Davies et al., 2004; Wessels and Perez-Pomares, 2004; Hohenstein and Hastie, 2006). Gain-of-function and loss-of-function approaches have been used to investigate the role of WT1 and its effect on EMT marker expression and cancer cell migrations. Silencing of *WT1* has been demonstrated to reduce proliferation, chemotaxis and invasiveness of human malignant mesothelioma cell lines (Plones et al., 2017). In cultured ovarian cancer cells and xenograft mouse models, *WT1* depletion significantly reversed EMT, inhibited cell migration and invasion, and prevented metastasis of cancer cells (Han et al., 2020).

**FIGURE 1:**
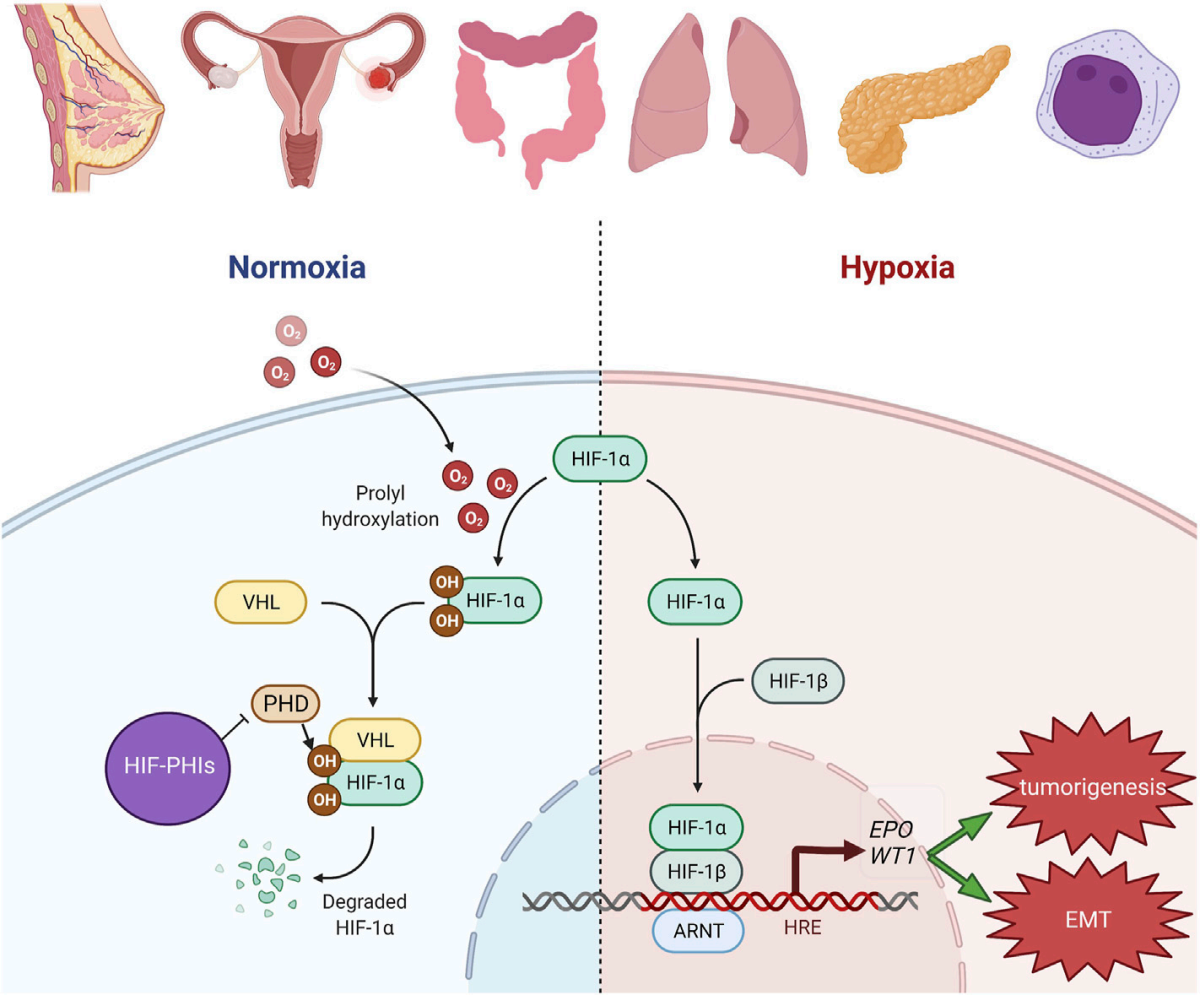
Hypothetic roles of WT1 in the development and progression of cancers with HIF pathway activation. In breast, uterine, ovarian, colon, lung, pleural, pancreatic and hematologic malignancies, HIF pathway is known to be activated especially in the hypoxic milieu. HIF-PHIs inhibit PHD catalysis and stabilize more HIF. The increased HIF resulted from either condition will bind to HRE and then transcriptionally activate its downstream genes, including *EPO* and *WT1*. Upregulation of *WT1* will contribute to tumorigenesis and EMT. The figure was created with BioRender.com.

An important observation is that EMT tends to occur in a hypoxic microenvironment. Exposure of breast cancer cells to a low-oxygen microenvironment facilitates cell migration by inducing the upregulation of vimentin and downregulation of epithelial marker proteins (Lester et al., 2007). This evidence collectively suggests the crucial roles of HIFs and WT1 in modulating EMT in cancer hypoxia. The hypothetical roles of WT1 in the development and progression of cancers with the activated HIF pathway are depicted in Figure 1. Although the pathogenesis of each cancer in Figure 1 could be more complex than depicted, this figure aims to highlight the link between HIFs and WT1 in these cancers.

## WT1 and Anemia

The exact mechanism by which WT1 benefits anemia remains largely unknown. It has been demonstrated that *Wt1* is required for the differentiation of the red blood cells. In conditional *Wt1* knockout mice, diminished extramedullary hematopoiesis within the red pulp compartment of the spleen was found. In addition, the *Wt1*-mutant bone marrow cells failed to differentiate into the erythrocyte lineage (Chau et al., 2011). In genetically manipulated cultured cell models, WT1 was shown to be the transcriptional activator of the *EPO* gene (Dame et al., 2006). Recently, by using *WT1* conditional knockout mice, Ji et al*.* demonstrated that WT1 recruits Tet2 to the promoter of EPO, which results in enhanced 5-hydroxymethylcytosine levels and the promotion of EPO expression (Ji et al., 2021). These important findings shed light on the potential beneficial role of WT1 in anemia.

## Strategies to Target WT1 to Avoid Cancer Development While Using HIF-PHIs to Treat Anemia

Currently available data from clinical trials on HIF-PHIs do not show cancer occurrence. In the phase II study of vadadustat in CKD patients, there were no reports of cancer (Pergola et al., 2016). In a study of 252 patients with non-dialysis CKD and 216 patients under dialysis treated with daprodustat, no malignancies were observed during the study (Holdstock et al., 2019). Recent data of large clinical trials on roxadustat have not shown the development of cancer (Chen et al., 2019). However, all these clinical studies were performed for less than 26 weeks. Long-term observations in humans will be required to examine the cancer-related risks of HIF-PHIs. Therefore, the first step of current strategies for using HIF-PHIs is carefully monitoring the occurrence of HIF-related cancers. As listed in Table 1, attention needs to be paid to the development of breast, lung, colorectal, pancreatic and hematologic malignancies. In addition, as *WT1* is one of the HIF downstream oncogene targets, it will be mandatory to monitor the development and progression of WT1-mediated cancers including ovarian, breast, lung, uterine, colon cancers, pleural mesothelioma and hematologic malignancies. In addition to monitoring, more research into WT1 inhibition in these cancers is required. WT1 peptide vaccine is known to induce clinical responses in MDS, AML, CML, ALL, multiple myeloma and various types of solid tumors including lung and breast cancers (Oka et al., 2004; Oka et al., 2017). Further investigations on the efficacy and safety of the WT1 peptide vaccine in other WT1-related cancers are required. Besides, vorinostat and bortezomib have been reported to significantly inhibit *WT1* gene expression in MO7-e and P39 cell lines, which are *in vitro* models for leukemia and MDS, respectively (Galimberti et al., 2008). In addition, curcumin is reported to decrease WT1 expression in patients’ leukemic cells (Anuchapreeda et al., 2006). Recently, the deubiquitinase inhibitor degrasyn was reported to promptly induce the degradation of endogenous and exogenous *WT1* in pancreatic ductal adenocarcinoma (Li et al., 2020). However, the therapeutic potential and the underlying mechanisms of these agents are yet to be investigated in ovarian, breast, lung, uterine, pancreatic, colon cancers, pleural mesothelioma and hematologic malignancies. More investigations are also required to examine if WT1 inhibition reduces the survival effects of HIF-PHIs in these cancers.

## Conclusion and Perspectives

HIF-PHIs activate HIF transcription factors, leading to an increase in endogenous EPO production and modulation of iron metabolism. Data on clinical trials has demonstrated their efficacy and short-term safety. HIF-PHIs have the potential to revolutionize the treatment of anemia in CKD but careful monitoring of the development or progression of cancer is required. Despite the persuasive links between hypoxia, HIF pathways, EMT and high levels of WT1 expression being observed in solid tumors, it is still yet to be answered in full whether WT1 is necessary or its overexpression alone is sufficient to drive tumorigenesis in human. In the coming era of using HIF-PHIs in renal anemia, a better understanding of the link between HIF and WT1 will help focus on the specific types of cancers to be monitored. In addition, more research on WT1 inhibition in ovarian, breast, lung, uterine, pancreatic, colon cancers, and pleural mesothelioma will contribute to the treatment of HIF-PHI-induced WT1-mediated cancers.
